# What Have We Learned? Implementation of a Shared Learning Agenda and Access Strategy for the Hormonal Intrauterine Device

**DOI:** 10.9745/GHSP-D-21-00789

**Published:** 2022-10-31

**Authors:** Kate H. Rademacher, Tabitha Sripipatana, Kendal Danna, Deborah Sitrin, Aurélie Brunie, Katie M. Williams, Kayode Afolabi, Francia Rasoanirina, Saumya Ramarao, Anne Pfitzer, Devon Cain, Morgan Simon, Elaine Menotti, Anna Hazelwood, Anthony Adindu Nwala, Zainab Saidu, Raveena Chowdhury, Anne Taiwo, Agnes Chidanyika, Gathari Ndirangu, Markus J. Steiner, Marie Chantale Lepine, Rick Homan, Abdulmumin Saad, John Vivalo, Laneta J. Dorflinger

**Affiliations:** a FHI 360, Durham, NC, USA.; b United States Agency for International Development, Washington, DC, USA.; c Population Services International, Washington, DC, USA.; d Jhpiego, Washington, DC, USA.; e Formerly of the Reproductive Health Division, Federal Ministry of Health, Abuja, Nigeria.; f Population Council, New York, NY, USA.; g Clinton Health Access Initiative, Boston, MA, USA.; h Global Health Supply Chain Program-Procurement and Supply Management project, Washington, DC, USA.; i Formerly of the Foreign, Commonwealth & Development Office; Now with Clinton Health Access Initiative, Monrovia, Liberia.; j Society for Health, Abuja, Nigeria.; k Clinton Health Access Initiative, Abuja, Nigeria.; l Marie Stopes International, London, United Kingdom.; m Marie Stopes International Nigeria, Abuja, Nigeria.; n United Nations Population Fund, New York, NY, USA.; o Formerly of United States Agency for International Development; Now with Bill & Melinda Gates Foundation, Washington, DC, USA.

## Abstract

In 2015, a global learning agenda for the hormonal intrauterine device (IUD) was developed with priority research questions regarding use of the method in low- and middle-income countries. In addition, members of the Hormonal IUD Access Group aligned on a strategy to expand access in the context of volunteerism and contraceptive method choice. This article synthesizes evidence generated since then and describes steps taken to address demand- and supply-side barriers to access. Findings demonstrated high continuation rates and satisfaction among hormonal IUD users that are comparable to other long-acting reversible contraceptives (LARCs). Across studies, a sizable number of users reported they would have chosen a short-acting method or no method at all if the hormonal IUD were not an option, which suggests that women did not see the hormonal IUD as interchangeable with other LARC options and thus it may fill an important niche in the market. With several countries now poised to scale up the method, resource mobilization will be key. On the demand side, investments in implementation research will be critical to understanding how best to launch and scale the method, while ensuring the sustainability of multiple quality-assured suppliers with affordable public-sector pricing will be necessary on the supply side.

## Abstract (precis)

Early evidence on clients’ and providers’ experiences with the hormonal intrauterine device in sub-Saharan Africa and lessons learned from implementing a shared learning agenda can inform strategies to expand access in other low- and middle-income countries.

## Teaser

**Key Findings:**

In this review of early experiences providing the hormonal intrauterine device (IUD) in sub-Saharan Africa, we found:

- Continuation and satisfaction were high among hormonal IUD users in pilot settings and generally comparable to those of other long-acting reversible contraceptives.
- Hormonal IUD users reported positive attributes of the method including its effectiveness, long duration, convenience, potential for reduced bleeding, and fewer side effects compared to other hormonal methods.
- Coordination across diverse organizations, including alignment on a shared learning agenda and access strategy, ultimately contributed to expanded access to the method.

**Key Implications:**

- As several countries in sub-Saharan Africa are preparing to introduce the hormonal IUD on a wider scale, the addition of the hormonal IUD to the catalogs of the United States Agency for International Development and the United Nations Population Fund will help expand public-sector access.
- Additional investments in method introduction—including provider training, demand creation, robust implementation research, and monitoring and evaluation—will be critical to understanding how best to scale the method.

## BACKGROUND

In 2015, an interagency working group convened with the goal of expanding access to the hormonal IUD (Box 1) in low- and middle-income countries (LMICs) in the context of volunteerism and contraceptive method choice.^1^^,^^2^ The hormonal IUD is a highly effective, long-acting reversible contraceptive (LARC) with proven noncontraceptive benefits including treatment for menorrhagia and potential prevention of iron-deficiency anemia.^3^ Despite these advantages, the method has not been available at scale in LMICs because of access barriers including high commodity prices, service delivery constraints, and lack of understanding of potential demand in these markets.^4^

BOX 1 **Hormonal Intrauterine Device Terminology**

The following additional terms are commonly used to describe the hormonal intrauterine device (IUD):

- Levonorgestrel-releasing intrauterine system (LNG IUS)
- Hormonal IUS
- LNG IUD

To accommodate multiple products and new hormone-releasing IUDs under development, the World Health Organization adopted the term “hormonal IUD” as the recommended standardized nomenclature in 2021.^1^

However, new opportunities to expand access have emerged in recent years, and the method was added to the World Health Organization’s Essential Medicines List in 2015.^3^^,^^5^ Part of the mandate of the interagency working group was to create a shared learning agenda for the method and align on harmonized approaches to data collection as well as stakeholder engagement strategies.^2^ Since then, what progress has been made in answering questions about the method? How did the consortium, now known as the Hormonal IUD Access Group (Box 2), facilitate coordination at the global and country levels? What are the next steps to expand availability of quality-assured products as part of a comprehensive contraceptive method mix? In this article, we synthesize learnings from the past several years and provide updates from the group’s efforts to address both demand- and supply-side barriers to access.

BOX 2 **Structure of the Hormonal IUD Access Group**

The Hormonal IUD Access Group is a global consortium of governments, donors, researchers, manufacturers, procurement agencies, and service delivery groups that are collaborating to expand access to the hormonal intrauterine device (IUD) in low- and middle-income countries.

The Hormonal IUD Access Group comprises 3 subgroups:

1. A steering committee with representatives from donor and procurer groups that assume responsibility for supporting global supply- and demand-side strategies to increase access. This group also includes non-decision-making members who support analyses and participate as a Secretariat.
2. A partners group comprised of key stakeholders who support the introduction of the method through a targeted, phased strategy at the country level. Government leadership is key to these efforts. This group also facilitates opportunities for manufacturers to share technical expertise and resources.
3. An operations group that supports monitoring of market health, development of communications, identifying and tracking country needs, and supporting goals of steering committee and partners group meetings.

## IMPLEMENTATION OF A SHARED LEARNING AGENDA: THEN AND NOW

One of the goals of the Hormonal IUD Access Group was to achieve consensus among a diverse group of stakeholders—including donors, researchers, suppliers, and service delivery organizations—on priority research and evaluation questions regarding the market potential of the hormonal IUD in LMICs. Although the hormonal IUD is widely available in high-income countries and has been frequently evaluated in those settings,^6^^,^^7^ evidence related to its use in LMICs has been limited. In recognition of this, stakeholders wanted to better understand potential opportunities and challenges for broader method introduction. This was considered especially important because uptake of the copper IUD has historically been low in many countries in sub-Saharan Africa.^8^ Although copper and hormonal IUDs have some attributes in common—including similar insertion and removal techniques—their side effect profiles and bleeding patterns are notably different, and thus, experiences with the 2 methods may differ among both providers and clients.^9^^–^^11^ In addition, because use of contraceptive implants has increased substantially in LMIC markets in recent years,^12^ there was interest in better understanding potential uptake of the hormonal IUD since both methods are hormonal LARCs. Also, evidence in some contexts has shown higher continuation rates for the hormonal IUD compared to implants.^3^ As such, stakeholders want to document continuation rates among users of the 3 LARCs in LMIC settings.

As a first step, preliminary market assessments were conducted by governments and implementing partners in several countries.^13^^–^^16^ In addition, the Hormonal IUD Access Group developed a shared learning agenda that focused on 5 domains: (1) client demand; (2) demand generation and marketing; (3) service delivery; (4) noncontraceptive attributes; and (5) cost-effectiveness and pricing.^2^ Key objectives included understanding potential demand and uptake among different populations (e.g., those with high unmet need for family planning, nonusers of contraception, and switchers from other methods); documenting continuation and satisfaction rates of hormonal IUD users compared to other methods; assessing clients’ understanding of and reactions to method attributes; and documenting providers’ perceptions of the method. Overall goals of having a shared learning agenda included harmonizing data collection approaches, aligning donor investments, avoiding duplication, and applying insights across countries to inform future introduction efforts, such as considerations related to policy, training, and demand creation.

The learning agenda aimed to understand potential demand and uptake, document continuation and satisfaction rates, assess clients’ understanding of method attributes, and document providers’ experiences with the method.

Since the learning agenda was finalized, implementing partners, donors, and suppliers have met on a regular basis to share emerging data and to promote a coordinated approach to stakeholder engagement at both country and global levels.^17^ Table 1 summarizes research projects led by organizations involved in the Hormonal IUD Access Group.

**TABLE 1.** Organizations and Projects That Supported Implementation of a Shared Learning Agenda for the Hormonal IUD

Project Name and Description	Funder (Years)	Partner Organizations	References With Key Findings
The EECO project, led by Catalyst Global (formerly WCG Cares), supports the introduction of new and underutilized contraceptive technologies, including the hormonal IUD, in the context of method choice and volunteerism. The project produces roadmaps for product introduction which can be used to scale up access. Under EECO, PSI introduced the hormonal IUD in Zambia beginning in 2017 and Madagascar in 2018.	USAID (2013–2023)	Catalyst Global, PSI	Danna et al.^24^
The SIFPO-2 project was intended to increase access to and use of high-quality, affordable family planning and other health information, products, and services globally.	USAID		
In 2016, under the SIFPO-2 project, MSI and MSION supported expanded hormonal IUD provision in pilot settings in Nigeria.	(2016–2017)	MSI, MSION, FHI 360	Eva et al.^18^
PSI’s SIFPO-2 project introduced the hormonal IUD in Nigeria and Zimbabwe in 2017 in collaboration with SFH. (Note: Data from Zimbabwe are unpublished; findings not included in this summary article).	(2014–2021)	PSI, SFH	Same as for EECO project
MCSP focused on 25 high-priority countries to prevent child and maternal deaths. The program introduced and supported high-impact, sustainable reproductive, maternal, newborn and child health interventions in collaboration with ministries of health and other partners. MCSP supported the introduction of the hormonal IUD in Kenya starting in 2016 and in Zambia starting in 2017.	USAID (2014–2019)	Jhpiego	Sitrin et al.^20^
LEAP Initiative generated timely, actionable evidence to help determine if and how expanded access to the hormonal IUD could increase contraceptive use and continuation rates in sub-Saharan Africa. FHI 360 implemented the initiative in partnership with PSI, SFH Nigeria and Zambia, and Catalyst Global.	Bill & Melinda Gates Foundation (2017–2022)	FHI 360, PSI, SFH	Brunie et al.^21^ Brunie et al.^22^ Brunie et al.^23^ Brunie et al.^19^

Abbreviations: EECO, Expanding Effective Contraceptive Options; IUD, intrauterine device; LEAP, Learning about Expanded Access and Potential of the LNG-IUS; MCSP, Maternal and Child Survival Program; MSI, Marie Stopes International; MSION, Marie Stopes International Organisation Nigeria; PSI, Population Services International; SFH, Society for Family Health; SIFPO-2; Support for International Family Planning and Health Organizations; USAID, United States Agency for International Development.

We review evidence generated by research partners included in the Hormonal IUD Access Group (Table 1).^18^^–^^24^ This article is not a systematic or scoping review of the literature but rather a review of evidence generated by the organizations that were proactively responding to questions articulated in the shared learning agenda. At the outset, Hormonal IUD Access Group members committed to harmonizing data collection approaches when feasible. This included sharing draft data collection forms among working group members and aligning on approaches and wording when appropriate.^25^ In addition, group members collaborated to summarize results across projects and provided joint dissemination of findings to both global- and country-level stakeholders.^26^ Table 2 provides a summary of the designs of the studies conducted in both public and social marketing sectors, and Table 3 lists the learning agenda questions that were addressed in countries where research was conducted (i.e., Kenya, Madagascar, Nigeria, and Zambia). Additional research was published on hormonal IUD use in LMICs during this period (2015–2021)^27^^–^^29^; however, those results are not summarized here.

**TABLE 2.** Research Studies That Were Designed to Respond to the Shared Learning Agenda for the Hormonal IUD

**Project**	**Research Time Frame**	**Country**	**Participants at Baseline** ^a^	**Study Design**	**Geographic Region**	**Service Delivery Context**	**Project Overview**
EECO	2018–2020	Madagascar	N=242	Longitudinal prospective survey within 12 months of insertion	Mahajanga, Toamasina, Antsiranana, and Antananarivo	19 social franchise clinics	Private sector Robust demand generation including to youth Higher price for clients for hormonal IUD compared to other LARCs
	2018–2019	Zambia	N=166		Copperbelt and Muchinga	19 public sector clinics	Public sector Free product and services CHW support
SIFPO-2 (MSI)	2016–2017	Nigeria	N/A	Routine service data and supplemental hormonal IUD client data, in-depth interviews with providers	16 states	76 social franchise providers, 9 mobile outreach teams, 20 public-sector providers	Public and private sector Free product and services Included mobile outreach component
SIFPO-2 (PSI)	2017–2019	Nigeria	N=208	Longitudinal prospective survey within 12 months of insertion	18 states	40 social franchise clinics	Private sector Higher price for clients for hormonal IUD compared to other LARCs Limited CHW support
MCSP	2017–2019	Kenya	N=432^b^	Provider-led “enhanced” data collection from their clients, follow-up phone interviews, focus group discussions with providers	Kisumu and Migori counties	42 public sector clinics	Public sector Free product and services
	2017–2019	Zambia	N=754^b^		Eastern, Central, Southern, Luapula provinces	41 public sector clinics	Public sector Free product and services Focus on maternity services
LEAP	2018–2019	Nigeria	N=835^c^	Mixed methods, prospective cohort studies; in-depth interviews with providers; focus group discussions with adolescent girls/youth; market research among potential users and providers	18 states	40 social franchise clinics	Private sector Higher price for clients for hormonal IUD compared to other LARCs Limited CHW support
	2018–2019	Zambia	N=607^c^		Copperbelt and Muchinga	21 public sector clinics	Public sector Free product and service CHW support

Abbreviations: CHW, community health worker; EECO, Expanding Effective Contraceptive Options; IUD, intrauterine device; LARCs, long-acting reversible contraceptives; LEAP, Learning about Expanded Access and Potential; MCSP, Maternal and Child Survival Program; MSI, Marie Stopes International; PSI, Population Services International; SIFPO-2, Support for International Family Planning and Health Organizations.

a Number of participants in quantitative surveys at baseline.

b Baseline total includes hormonal IUD and copper IUD adopters.

c Baseline total includes hormonal IUD, copper IUD, and implant users. Longitudinal data were collected from 367 women.

**TABLE 3.** Shared Learning Agenda Questions That Were Addressed by Project^a^

**Shared Learning Agenda: 2015–2020**	**EECO**	**SIFPO2 (MSI)**	**SIFPO2 (PSI)**	**MCSP**	**LEAP**
	**Madagascar, Zambia**	**Nigeria**	**Nigeria, Zimbabwe**	**Kenya, Zambia**	**Kenya,** **Nigeria, Zambia**
A. Client demand					
1. What are the profile(s) of the clients who will use this product?	X^b^		X	X	X
a. Is there or would there be demand for this product among subpopulations with high unmet need for FP (e.g., women in lower wealth quintiles, PP women, adolescents, WLHIV, PAC clients)?	(x)^c^		(x)	(x)	(x)
b. Will introduction of the hormonal IUD help reach new FP users (i.e., current non-users)?	X		X	(x)	X
c. To what degree will introduction of the hormonal IUD result in “switching” and from what methods?	X		X	(x)	X
2. Does the hormonal IUD have the potential to “revitalize” the IUD market in FP2020 countries?					
a. Would demand for the hormonal IUD be higher than demand for the copper IUD has been?		(x)		(x)	(x)
3. Would introduction of the hormonal IUD increase FP use overall/increase contraceptive prevalence rate(s)?					
a. Can scale-up of this product help meet FP2020 goals?					
4. How do continuation rates of the hormonal IUD compare to continuation rates of other LARCs?	(x)		(x)	(x)	X
5. Does immediate postpartum access to the hormonal IUD increase use of postpartum FP overall?					
B. Marketing					
6. What are effective demand creation strategies with different populations and in different sectors?	(x)		(x)		(x)
7. Can promotion of FP including the hormonal IUD be integrated into other health sectors?				(x)	
C. Service delivery					
8. How can we overcome barriers that have impacted provision of copper IUD when introducing hormonal IUD?	(x)	(x)	(x)	(x)	(x)
9. What are health care providers' perceptions of this product?	X	X	X	X	X
10. What are effective service delivery models for hormonal IUD provision? How does it differ by context?	(x)	(x)	(x)	(x)	(x)
a. What are effective provider training strategies for the hormonal IUD?				(x)	
D. Noncontraceptive attributes					
11. How does knowledge of noncontraceptive attributes of the hormonal IUD affect uptake and use?	X	(x)	(x)		X
a. What noncontraceptive attributes are most attractive to women in different contexts?	X	(x)	(x)		X
b. What noncontraceptive attributes are seen as most beneficial by providers?	X	(x)	(x)	(x)	X
12. What are perceptions of amenorrhea among providers and various client segments?	(x)	(x)	(x)	(x)	X
13. Can scale-up of the hormonal IUD help reduce rates of anemia?					
E. Cost-effectiveness and pricing					
14. To what extent is the hormonal IUD cost-effective compared to other FP methods including other LARCs?					X
15. What is the willingness-to-pay for the hormonal IUD among different populations of clients/groups?					(x)

Abbreviations: EECO, Expanding Effective Contraceptive Options; FP, family planning; IUD, intrauterine device; LARCs, long-acting reversible contraceptives; LEAP, Learning about Expanded Access and Potential; MCSP, Maternal and Child Survival Program; MSI, Marie Stopes International; PAC, postabortion care; PP, postpartum; PSI, Population Services International; SIFPO-2, Support for International Family Planning and Health Organizations; WLHIV, women living with HIV.

a This table is intended to summarize evidence collected across countries and projects, but not all results are reflected in this synthesis paper. For additional results, refer to the Hormonal IUD Access Portal website at www.hormonaliud.org.

b X = data available.

c (x) = Some data collected but quantity/quality limited. Assessment of the current status of evidence based on review and assessment of existing data from each country and project.

All research conducted by members of the Hormonal IUD Access Group in pilot introduction settings, except in Madagascar, involved the use of product donated by the International Contraceptive Access (ICA) Foundation, a public-private partnership between the Population Council and Bayer AG (Box 3).^30^^–^^37^ In Madagascar, Population Services International and Catalyst Global (formerly WCG Cares) evaluated the introduction of Avibela, a new hormonal IUD product developed by Medicines360 that was approved for use there in 2018 (Box 4).^38^^–^^40^ Population Services International and Catalyst Global also supported the introduction of Avibela in Zambia starting in 2019; however, the research was initiated before Avibela introduction when the ICA Foundation product was still being used.

BOX 3 **Development of Mirena and Overview of the International Contraceptive Access Foundation**

Over the past 65 years, the Population Council has developed and introduced several of the world’s most effective and popular contraceptive methods, including the hormonal intrauterine device (IUD). The Population Council began developing the hormonal IUD in the late 1970s to create a long-acting reversible contraceptive that combined the beneficial features of both hormonal contraceptives and IUDs.

Mirena, the innovator product, was developed by the Population Council and Bayer AG (formerly Leiras Oy) and first approved in Finland in 1990. In 2000, Mirena was approved by the United States Food and Drug Administration (U.S. FDA), which expanded its approval to include treatment of heavy menstrual bleeding in 2009.^30^ Produced in Finland by Bayer AG, Mirena is now distributed in more than 120 countries worldwide. Clinical data demonstrate high efficacy, safety, and acceptance.^31^^,^^32^ The method has a pregnancy rate of less than 1% in the first year of use, which is comparable to sterilization, and has the ability to lighten menstrual bleeding, which can prevent the need for hysterectomies among women with menorrhagia.^33^ Originally approved for up to 5 years of use, Mirena’s contraceptive indication was extended for up to 8 years of pregnancy prevention in 2022 by the U.S. FDA.^34^

Although Mirena is available in more than 120 countries, it has been largely unavailable in low- and middle-income countries until recently. In 2003, the Population Council and Bayer AG collaborated to establish the International Contraceptive Access (ICA) Foundation, a public-private partnership, as a strategy to address limited access.^35^ The ICA Foundation donates an unbranded version of the product called the LNG IUS in low-resource settings outside of the United States (Table 4).

**TABLE 4.** Overview of Hormonal IUD Products Approved by a Stringent Regulatory Authority and Available in Low-Income Countries^a^

**Supplier**	**Quality-Assured Hormonal IUD Product Containing 52 mg LNG**
Bayer AG^b^	Mirena
International Contraceptive Access Foundation	Unbranded LNG IUS product
Medicines360	Avibela^c^

Abbreviation: IUD, intrauterine device; LNG, levonorgestrel; SRA, stringent regulatory authority.

a In addition to the products listed in the table, several hormonal IUD products are being introduced in a limited number of FP2020/FP2030 countries that are not currently approved by an SRA. In 2022, Mirena was the first hormonal IUD product to be prequalified by the World Health Organization.

b Bayer AG also manufactures the lower-dose hormonal IUD products Skyla and Kyleena. However, these products are not yet available in low- and middle-income countries, and therefore are not discussed in this paper.

c The Medicines360 product is sold in the United States under trade name Liletta; the product is sold in FP2020/FP2030 countries under the trade name Avibela.

As of early 2022, the ICA Foundation has donated more than 180,000 LNG IUS devices to organizations in 39 countries. These donations support the work of governments and local nonprofit organizations, hospitals, and global partners.^36^^,^^37^ Across multiple contexts, ICA Foundation donations have helped expand access to the method and laid the groundwork for broader hormonal IUD introduction. In several countries, including Kenya, Nigeria, and Zambia, donations supported demonstration projects that provided a basis for the inclusion of the hormonal IUD within national health systems and contributed to the government’s decision to scale up the method.

BOX 4 **Introduction of Avibela in Low- and Middle-Income Country Settings**

In 2015, the U.S. Food and Drug Administration approved a hormonal intrauterine device (IUD) product, Liletta, which was brought to market by Medicines360, a global nonprofit women’s health pharmaceutical company, to expand access to the method for women around the world.^38^ In the United States, Liletta is available to qualifying public health clinics at a discounted price, and Medicines360 engages with public sector clinics on an ongoing basis to help ensure they can obtain and maintain access.

The more affordable price of Medicines360’s hormonal IUD also created momentum around the consideration of the method as a viable option for women in low- and middle-income countries (LMICs). Through Medicines360’s subsidiary, Impact RH360, the product is being registered in LMICs under the trade name Avibela. Impact RH360 works with international nongovernmental organizations and pharmaceutical company partners to support registrations, provider training, and product introduction. As of early 2022, Avibela had been registered in 7 countries in sub-Saharan Africa with additional registrations pending.^39^ Avibela is currently approved for up to 6 years of use, and Medicines360 has plans to further extend the duration of use.^40^

## SYNTHESIS OF KEY LEARNINGS GENERATED ON THE HORMONAL IUD

We offer an overview of some of the key insights from the research led by organizations in the Hormonal IUD Access Group including a summary of similarities and differences observed across the studies. It is important to note that the contexts of the introduction programs influenced which client population(s) were reached with hormonal IUD services (Table 2), and this may therefore have influenced clients’ and providers’ experiences with the method. As such, results should be interpreted cautiously as they may not represent experiences if and when the method is made available on a wider scale.

### Client Demand

#### Demographics of Hormonal IUD Users

Most hormonal IUD users across pilot settings were married women aged older than 25 years with children and many had secondary education (Table 5). In both Madagascar and Kenya, a sizable minority of hormonal IUD users were aged younger than 25 years (30% and 41%, respectively). The Madagascar pilot involved demand generation in the private sector that included outreach to youth. Under the Maternal and Child Survival Program in Kenya and Zambia, where hormonal IUD services were offered in public-sector settings and providers were trained on postpartum insertion techniques, a substantial percentage of the insertions were among women in the postpartum period (Table 5).

**TABLE 5.** Demographics for Hormonal IUD Adopters

Project (N at baseline)	EECO (N=242)	EECO (N=166)^a^	SIFPO-2 (MSI) (N=286)^b^	SIFPO-2 (PSI) (N=208)	MCSP (N=289)^c^	MCSP (N=359)^c^	LEAP (N=266)^d^	LEAP (N=153)^d^
	Madagascar	Zambia	Nigeria	Nigeria	Kenya	Zambia	Nigeria	Zambia
Age, years, %								
<25	30.0	17.0	See below^e^	6.0	41.0	21.0	8.0	22.0
25+	70.0	82.0		94.0	55.0	72.0	92.0	78.0
Married, %	79.0	84.0	93.8	95.0	85.8	83.0	97.0	78.0
Mean parity	1.7	3.3	4.4	3.3	N/A^f^	4.1	3.3	3.0
Secondary education or higher, %	82.0	52.0	69.5	89.0	57.0	45.0	86.0	50.0
Timing of insertion, %^g^								
Postpartum (<48 hours)					8.7	27.9		
Postpartum (48 hours to 1 year)					52.6	30.1		
Postabortion					0.4	4.3		
Not post-pregnancy					37.4	36.2		
Missing					1.0	1.5		

Abbreviations: EECO, Expanding Effective Contraceptive Options; IUD, intrauterine device; LEAP, Learning about Expanded Access and Potential; MCSP, Maternal and Child Survival Program; MSI, Marie Stopes International; PSI, Population Services International; SIFPO-2, Support for International Family Planning and Health Organizations.

a While 166 respondents participated in the baseline and 155 respondents participated in the follow-up, not all respondents answered all survey questions. As a result, in Zambia proportions will not always equal 100%.

b Sociodemographic data available for 30% of hormonal IUD clients (n=286) although not all women answered all of the related questions.

c Study includes hormonal and copper IUD adopters, but table only includes hormonal IUD adopters.

d Study includes hormonal, copper IUD, and implant adopters, but table only includes hormonal IUD adopters.

e Of the available sociodemographic data for hormonal IUD adopters, the mean age was 34 years.

f Parity collected as categorical variable so mean parity not available. 49% of hormonal IUD adopters had parity 1–2, 30% had parity of 3–4, 15% had parity of 5+, and 4% had parity of 0.

g Data for “timing of insertion” for Kenya came from 289 hormonal IUD adopters; for Zambia, it came from 395 hormonal IUD adopters.

#### Previous Methods Used

Most hormonal IUD users had previously used other modern contraceptive methods; however, between 5%–23% of adopters were new family planning users across the settings. Among those who were prior contraceptive users, the majority in most studies reported using a short-acting method as their last method before uptake of the hormonal IUD (data not shown).^20^^–^^24^

#### Continuation Rates

Cumulative continuation rates for the hormonal IUD were consistently high, ranging from 81%–95% at 12 months.^20^^,^^21^^,^^24^ In a study that compared hormonal IUD, copper IUD, and implant users in Nigeria and Zambia,^21^ the 12-month cumulative continuation rates were 87% in Nigeria and 95% in Zambia for the hormonal IUD, 87% in Nigeria and 89% in Zambia for the copper IUD, and 85% in Nigeria and 83% in Zambia for implants (Table 6).

**TABLE 6.** Continuation Rates for Those Studies That Followed Clients Over Time

Time Frame	6 Months		12 Months				
Project	LEAP		EECO		SIFPO-2 (PSI)	LEAP	
	Nigeria, % (95% CI)	Zambia, % (95% CI)	Madagascar, % (95% CI)	Zambia, % (95% CI)	Nigeria, % (95% CI)	Nigeria, % (95% CI)	Zambia, % (95% CI)
Hormonal IUD	n=266	n=140	n=242	n=166	n=208	n=266	n=140
	94.3 (90.7, 96.5)	96.3 (91.3, 98.4)	84.0 (76.0, 90.0)	81.8 (74.7, 87.1)	90.0 (80.7, 94.9)	86.8 (82.1, 90.4)	94.7 (89.2, 97.4)
Copper IUD	n=274	n=149				n=274	n=149
	92.4 (88.5, 95.0)	94.0 (88.4, 97.0)				86.9 (82.1, 90.4)	89.1 (82.3, 93.4)
Implant	n=295	n=78				n=295	n=78
	91.6 (87.7, 94.3)	90.4 (80.9, 95.3)				85.0 (80.2, 88.7)	83.1 (72.2–90.1)

Abbreviations: CI, confidence interval; EECO, Expanding Effective Contraceptive Options; IUD, intrauterine device; LEAP, Learning about Expanded Access and Potential; PSI, Population Services International; SIFPO-2, Support for International Family Planning and Health Organizations.

Cumulative continuation rates for the hormonal IUD were consistently high, ranging from 81%–95% at 12 months.

#### Satisfaction Rates and Perceived Positive Method Attributes

Satisfaction with the hormonal IUD was generally high with 80%–98% reporting being satisfied or very satisfied with method use across the time points when data were collected.^20^^–^^24^ The study in Nigeria and Zambia that included copper IUD and implant users also documented high satisfaction rates with other LARCs (100% in Nigeria and 98% in Zambia for the copper IUD, and 95% in Nigeria and 90% in Zambia for implants).^21^ Positive method attributes most commonly reported by hormonal IUD users included effectiveness, long duration, convenience, reduced bleeding, and fewer side effects (data not shown).^20^^–^^24^

### Demand Generation and Marketing

#### Sources of Information About the Method

Except for Madagascar where 70% of hormonal IUD users heard about the method from an outreach worker, providers were the main source of information about the hormonal IUD (data not shown).^20^^–^^24^

#### Reasons Hormonal IUD Users Chose the Method

Across study settings, users mentioned long duration as a top reason they chose the hormonal IUD. Other common reasons included effectiveness, potential for fewer side effects compared to other hormonal methods, reduced bleeding with use of the method, convenience, recommended by others, and ability to use discreetly (data not shown).^20^^–^^24^

#### What Clients Would Have Done if the Hormonal IUD Was Not Available

Across studies, between 16%–54% of hormonal IUD users reported they would have chosen a short-acting method if the hormonal IUD were not an option. Also, between 3%–46% said they would have chosen no method if the hormonal IUD was not available, with rates highest in Madagascar and Nigeria (data not shown).^20^^–^^24^

#### Potential Demand Among Adolescent Girls and Young Women

In focus group discussions conducted in Nigeria and Zambia with adolescent girls aged 15–19 years,^41^^,^^42^ positive method attributes most commonly mentioned by respondents in both countries included the potential for reduced or no menstrual bleeding and the potential for reduced menstrual cramps/pain. In both countries, perceptions of amenorrhea were mixed, with some respondents feeling it would primarily be an advantage, others feeling it would primarily be a disadvantage, and others mixed in their feelings. When asked about the insertion procedure for the hormonal IUD, some respondents in both countries expressed concern that the location is too intimate or that insertion would be painful. Others felt that the insertion procedure would not be a problem and liked that the method can be used discreetly.

### Service Delivery

Qualitative data on providers’ experiences indicated that across settings providers generally appreciated the method’s therapeutic benefits, especially reduction of heavy bleeding and treatment of menstrual disorders.^18^^–^^20^ Providers across most settings had previous experience with LARCs (the Maternal and Child Survival Program in Zambia was an exception; it focused on LARC trainings in maternity settings with inexperienced providers). Almost all providers who were interviewed felt comfortable with hormonal IUD provision after training. Lack of awareness of the hormonal IUD as an option among women was perceived as a key barrier to uptake.

### Noncontraceptive Attributes

Data from Kenya, Nigeria, and Zambia on perceptions and experiences with menstrual changes showed that most women who experienced reduced bleeding and/or amenorrhea with hormonal IUD use reported it had a positive impact on their lives.^18^^,^^20^^–^^24^ In addition, results from a study in Nigeria and Zambia found that hormonal IUD users reported needing fewer menstrual products to manage their periods compared to before they began using the method relative to copper IUD or implant users.^21^

### Cost-Effectiveness and Pricing

A cost-effectiveness analysis comparing LARC options was conducted based on continuation data from research conducted in Nigeria and Zambia as well as data from the respective Demographic and Health Surveys.^43^ Costs were estimated using method-specific Markov models considering the probability of method switching and method failure for 1,000 simulated cohorts of 100,000 women of reproductive age in both countries. Results showed that over a 10-year period, the hormonal IUD was more cost-effective (i.e., lower incremental cost per unintended pregnancy avoided) compared to implants from both a health systems and societal perspectives, with the copper IUD remaining the most cost-effective LARC option over the 10-year period.

Results showed that over a 10-year period, the hormonal IUD was more cost-effective compared to implants from both a health systems and societal perspectives.

## MOVING FROM INTRODUCTION TO SCALE

Results from the research findings were disseminated in the countries where pilot projects were conducted, as well as among global audiences. Based on emerging evidence, increased affordability and availability of quality-assured products, and a desire to expand contraceptive method choice, governments in several countries have decided to scale up the hormonal IUD in the public sector. Development of costed introduction plans are underway.^44^ Boxes 5 and 6 include case studies from Nigeria^45^^–^^50^ and Madagascar,^51^^,^^52^ respectively.

BOX 5 **Development of a National Introduction Plan in Nigeria**

Nigeria is the most populous country in Africa, with an estimated 45 million women of reproductive age.^45^ In 2018, Nigeria’s modern contraceptive prevalence rate was 12% among married women, reflecting an increase from 10% in 2013.^46^ Injectables and implants are the most popular methods with 23% and 24% of modern contraceptive users choosing these, respectively. In contrast, the copper intrauterine device (IUD) comprises 6% of the modern method mix.^47^ Nearly 80% of copper IUDs are provided through the public sector, with 40% obtained from government hospitals.^45^

The federal government of Nigeria has prioritized family planning as a core program under the essential package of health care services in the National Strategic Health Development Plan II which provides a 5-year roadmap for Nigeria’s health sector.^48^ The stated goal under the Nigeria Family Planning Blueprint (2020–2024) is to increase the modern contraceptive prevalence rate to 27%.^49^

Until recently, Nigeria’s strategy for long-acting reversible contraceptives focused on scaling up access to implants and the copper IUD. The hormonal IUD is yet to be widely available in the public sector, although several groups have supported early introduction of the method, including Rotary International which began offering the method in 2007.^50^ As noted above, results from pilot studies in Nigeria revealed high continuation and satisfaction rates among users. Findings also suggested that women who are counseled on a full range of methods may perceive that the hormonal IUD has unique benefits. After the dissemination of research results, in 2021, the Federal Ministry of Health (FMOH) adopted a national plan to introduce and scale up the hormonal IUD.

Members of the Product Introduction Coordination Mechanism (PICM) of the national Reproductive Health Technical Working Group led the development of the plan, which calls for phased implementation focused around 5 main thematic areas:

1. **Coordination**: The PICM will facilitate resource mapping as well as harmonization of partners’ introduction efforts.
2. **Capacity building**: Preservice curricula will be updated by partners and a comprehensive plan for training in-service providers will be created and rolled out.
3. **Procurement and supply chain management**: The FMOH will support adding the hormonal IUD to the national Essential Medicines List and guide the national quantification process and procurement of hormonal IUD commodities.
4. **Demand generation and communication**: Leveraging existing demand generation activities conducted by states and partners, it is recommended that messages be included to increase awareness and promote uptake of the hormonal IUD in the context of volunteerism. The method will also be added to existing and new counseling materials.
5. **Monitoring and supervision**: National health information management systems are being modified to include the hormonal IUD and being used to report on use of the method. Reporting and monitoring will feed into existing data and supervision systems.

BOX 6 **Efforts to Expand Access to Hormonal Intrauterine Device in Madagascar**

The modern contraceptive prevalence rate among married women in Madagascar is 43% and 35% among all women. The rate of unmet need for contraceptive methods remains high at 22% among married women. The method mix is heavily skewed toward injectables (66% of contraceptive users).^51^

To help expand method choice, Population Services International (PSI) Madagascar supported the Ministry of Public Health (MOPH) to begin planning for the introduction of the hormonal intrauterine device (IUD) as part of a comprehensive method mix; the method was added to the national family planning guidelines in 2018.

After that, 28 private providers in PSI Madagascar’s social franchise network and 19 private providers outside this network were trained on the hormonal IUD and certified by the MOPH, and PSI Madagascar began offering the method at 30 private health facilities with funding from the United States Agency for International Development through the Expanding Effective Contraceptive Options project. As previously noted, research carried out at these facilities demonstrated high continuation after 12 months. The research also highlighted the broad appeal of the hormonal IUD across demographic profiles; these findings informed a broader marketing strategy for the method. In addition, provider champions were engaged to increase awareness of the method.

PSI collaborated with the MOPH to share these findings, and the MOPH then began developing a plan to scale up the method in the public sector. As of mid-2021, the MOPH and PSI began introducing the hormonal IUD in 6 of Madagascar's 22 public sector regions with support from the Catalytic Opportunity Fund—a mechanism managed by the Clinton Health Access Initiative with support from the Government of the United Kingdom Foreign, Commonwealth, and Development Office and other donors—which provides funding to enable product introduction activities including for the hormonal IUD.^52^ As part of the initiative, master trainers will provide cascade trainings in public sector facilities, and the hormonal IUD will be included in campaigns led by community educators to generate awareness.

At the same time, the Hormonal IUD Access Group made substantial strides in addressing supply-side barriers to access. In 2021, the hormonal IUD was added for the first time to the United States Agency for International Development (USAID) and United Nations Population Fund (UNFPA) product catalogs (both the Mirena and Avibela products).^53^ To facilitate distribution in LMICs, USAID’s family planning priority countries are eligible to procure the product through the USAID Global Health Supply Chain Program-Procurement and Supply Management project. The method is also available for procurement through UNFPA to all countries participating in the UNFPA Supplies Partnership or other countries using domestic or other resources.

## LESSONS LEARNED FROM THE HORMONAL IUD ACCESS GROUP COORDINATION PLATFORM

In a previous assessment of the initial interagency working group, the advantages and disadvantages of the global coordination platform were assessed.^2^ Here, we expand on these insights with a summary of some of the activities and lessons learned from the experiences of the Hormonal IUD Access Group.

### A Shared Learning Agenda Improved Harmonization and Achievement of Mutual Research Objectives

The coordinated approach generated a body of data that catalyzed progress at the country and global levels. Research activities were intended to answer learning questions in both public-sector and social-marketing settings with a focus on user perspectives and experiences. Results helped inform governments’ decision making about if and when to scale up the method. The shared learning agenda also facilitated coordination between and within donors. Often donor organizations have multiple managers making funding decisions in different areas including service delivery, research, and commodity procurement; the learning agenda allowed for greater coordination to identify priorities and investment gaps.

A shared learning agenda across diverse organizations improved harmonization and allowed for targeted support to achieve mutual research objectives.

However, implementing research within donor-supported projects created some limitations in achieving globally applicable insights. All early introduction and research activities included in the Hormonal IUD Access Group were based in sub-Saharan Africa; the locations selected depended on where nongovernmental organization partners had country capacity, where ministries of health were interested, and where donor country offices provided approvals for activities. Also, working with a tightly defined group of partners with specific donor funding limited outreach to some organizations in other geographic regions such as recipients of ICA Foundation donations based in South America^54^; this is a shortcoming that should be addressed moving forward.

### The Hormonal IUD Access Group Facilitated Stakeholder Engagement and Alignment on an Introduction Strategy

Ongoing coordination through the Hormonal IUD Access Group facilitated engagement with suppliers and led to alignment around a phased, focused introduction strategy that addresses demand- and supply-side considerations. As early introduction efforts progressed, additional governments, partners, and donors became engaged, which in turn, accelerated momentum. In 2019–2020, stakeholders aligned on an updated strategy with the shared goals of (1) ensuring availability of affordable, quality-assured products to facilitate sustainable markets; and (2) supporting countries that are ready to introduce and scale up the method through a phased and focused approach. The current structure of the Hormonal IUD Access Group (Box 2) has allowed diverse stakeholders to feed into this strategy. Coordination across procurers and country governments is intended to ensure that demand for the product does not outstrip supply. Concurrently, there are continued efforts to manage supply security, accessibility, and affordability over the long term.

The Hormonal IUD Access Group has allowed diverse stakeholders to align on shared goals of ensuring availability of affordable products and supporting countries to introduce and scale up the method.

Also, by including a subgroup of implementing partners, the Hormonal IUD Access Group facilitates knowledge exchange within and between specific projects and countries. This means the Steering Committee’s decision making is informed by country-level experiences and government priorities, which has helped to reduce inefficiencies and avoid duplication. In addition, the operating structure has allowed the group to effectively collaborate with hormonal IUD suppliers, thus providing assurances regarding the global community’s vision and planning. This has been an important enabling factor underpinning manufacturer engagement and facilitation of public-sector access strategies.

However, there are limitations to method-specific introduction efforts. Other recent coordination mechanisms have also focused on specific methods (e.g., the Implant Access Group and DMPA-SC Access Group). Although these groups have been effective in helping expand access,^55^^,^^56^ their method-specific approaches may constrain countries’ ability to plan for product introduction across method categories and may impede cross-cutting resource mobilization. Siloed introduction efforts may also contribute to product introduction fatigue among stakeholders at the country level.^57^ Moving forward, a key question is how to balance cross-method coordination with method-specific considerations. New initiatives such as Shaping Equitable Market Access for Reproductive Health (SEMA), launched in 2021, may help address these challenges.^58^

### Achieving More Affordable Hormonal IUD Public-Sector Pricing Was Not Based on a Volume Guarantee

Current hormonal IUD public-sector pricing does not depend on donor-driven market shaping interventions. Experience shows that market-shaping interventions can help expand access to critical health care products including contraceptives.^59^ Recent efforts to expand access to contraceptive implants and injectables have been supported through donor-backed minimum volume guarantees. These interventions helped to lower pricing in the public and social marketing sectors and were therefore key to facilitating rapid scale-up in LMIC markets. The lower unit cost achieved through the Implants Access Program was one of the primary reasons for a tenfold increase in implant procurement between 2010 and 2018.^55^ Similarly, broader introduction of a subcutaneous depot medroxyprogesterone acetate injectable was achieved when pricing agreements for procurement in LMICs were negotiated with the manufacturer.^60^ However, while these strategies have helped increase access to both methods, they have also posed challenges. In some cases, the sustainability of supply is uncertain. Also, procurement volumes needed to achieve lower public-sector pricing have been based on global volume guarantee targets, rather than being driven primarily by countries’ health system capacity or demand. In contrast, the Hormonal IUD Access Group does not have a mandate to meet specific volume targets; rather, the group is focused on supporting country-level decision making regarding if and when to expand their method mix to include the hormonal IUD and on facilitating careful supply management across countries. Moving forward, it will be important to conduct further analyses related to the impact of these different approaches, including on the pace of product introduction and sustainability of pricing and supply.

## THE WAY FORWARD

Results from research activities and early introduction efforts have addressed preliminary questions about the potential of the hormonal IUD in markets in sub-Saharan Africa. Findings have demonstrated high continuation rates and satisfaction among hormonal IUD users that are comparable to other LARCs. Across studies, a sizable number of users reported they would have chosen a short-acting method or no method at all if the hormonal IUD were not an option. These findings suggest that women did not see the hormonal IUD as interchangeable with other LARC options and thus that the method may fill an important niche in the market; these findings are consistent with results from an earlier study in Kenya.^61^^,^^62^ Early evidence also suggests that the method may appeal to women in different demographic groups including adolescent girls, although uptake among younger women may remain lower without targeted outreach.^24^^,^^41^^,^^42^

It is notable that the potential for fewer side effects was a common reason that women chose the hormonal IUD across settings. Because the hormonal IUD involves localized release of levonorgestrel, it can result in lower systemic blood levels relative to other hormonal methods which may mean side effects are less pronounced for some women.^3^ Potential users seemed to understand this point after counseling, a finding which was also reflected in other qualitative research in Kenya among early adopters of Mirena.^63^ This finding is important since the most common reason women in LMICs cite for not using and discontinuing contraception are side effects.^64^ Results also indicated that the potential for reduced bleeding was a common reason women chose the method across settings. Data suggest that both women and providers appreciate the noncontraceptive attributes of the method, especially reduction of heavy bleeding and treatment of menstrual disorders. Women who experienced reduced or amenorrhea (paused bleeding) with the hormonal IUD generally reported that it had a positive impact on their lives overall, although perceptions of amenorrhea were more mixed, with fears remaining that amenorrhea can harm one’s health or fertility. Given this, effective counseling on contraceptive-induced menstrual changes remains a priority.^65^^,^^66^

Data suggest that both women and providers appreciate the noncontraceptive attributes of the method, especially reduction of heavy bleeding and treatment of menstrual disorders.

Globally, the addition of the hormonal IUD in the USAID and UNFPA product catalogs for the first time in 2021 is a major milestone that will help expand public-sector access in LMICs. Several countries are now poised to scale up the method and have or are in the process of developing national introduction plans and forecasting for initial commodity needs. Moving forward, resource mobilization will be key. There is some identified funding to cover initial introduction efforts, but additional investments from governments and the private sector will be critical. In addition, in 2021, members of the Hormonal IUD Access Group in consultation with country governments refreshed the global learning agenda to reflect priority research questions in this new phase (Supplement). Additional investments in implementation research and robust monitoring and evaluation will be critical to understanding how best to launch and scale the method including how to raise awareness among potential users, ensure high-quality counseling and care in various settings, and address current and potential barriers and enablers for wider availability and use.

Addressing supply-side issues will also be important moving forward; having multiple quality-assured suppliers with affordable public-sector pricing is key for the long-term health of global markets. The Hormonal IUD Access Group and other partners will continue to work with suppliers to facilitate and monitor product availability and affordability. On the service delivery side, training providers and generating awareness of and demand for the hormonal IUD will be an ongoing priority, especially since provider- and client-side barriers have often limited uptake of the copper IUD.^8^ There are also opportunities to support integrated service delivery and increase connections with other health areas including maternal health, nutrition, and menstrual health.^67^ Research is ongoing to further evaluate the potential of the hormonal IUD to help alleviate anemia.^68^ Continued coordination to address these priorities will help maximize impact as the hormonal IUD is introduced at a wider scale.

## Supplementary Material

GHSP-D-21-00789-supplement.pdf

## Notes

**Peer Reviewed**

**First published online:** October 5, 2022.

## References

[1] World Health Organization (WHO). *WHO Statement on Levonorgestrel-Releasing Intrauterine Device Nomenclature*. WHO; 2021. Accessed August 7, 2021. https://www.who.int/publications/i/item/9789240021730

[2] Rademacher KH Sripipatana T Pfitzer A. A global learning agenda for the levonorgestrel intrauterine system (LNG IUS): addressing challenges and opportunities to increase access. Glob Health Sci Pract. 2018;6(4):635–643. 10.9745/GHSP-D-18-00383. 30591573 PMC6370355

[3] Hubacher D. The levonorgestrel intrauterine system: reasons to expand access to the public sector of Africa. Glob Health Sci Pract. 2015;3(4):532–537. 10.9745/GHSP-D-15-00178. 26681701 PMC4682579

[4] Jacobstein R Shelton JD. The levonorgestrel intrauterine system: a pragmatic view of an excellent contraceptive. Glob Health Sci Pract. 2015;3(4):538–543. 10.9745/GHSP-D-15-00330. 26681702 PMC4682580

[5] Rademacher KH Solomon M Brett T. Expanding access to a new, more affordable levonorgestrel intrauterine system in Kenya: service delivery costs compared with other contraceptive methods and perspectives of key opinion leaders. Glob Health Sci Pract. 2016;4(Suppl 2):S83–S93. 10.9745/GHSP-D-15-00327. 27540128 PMC4990165

[6] Hatcher RA Kowal D Nelson AL. *Contraceptive Technology*. 21st ed. Ayer Company Publishers, Inc.; 2018.

[7] Peipert JF Zhao Q Allsworth JE. Continuation and satisfaction of reversible contraception. Obstet Gynecol. 2011;117(5):1105–1113. 10.1097/AOG.0b013e31821188ad. 21508749 PMC3548669

[8] Benova L Cleland J Daniele MAS Ali M. Expanding method choice in Africa with long-acting methods: IUDs, implants or both? Int Perspect Sex Reprod Health. 2017;43(4):183–191. 10.1363/43e5217. 29874164

[9] Bahamondes L Fernandes A Monteiro I Bahamondes MV. Long-acting reversible contraceptive (LARCs) methods. Best Pract Res Clin Obstet Gynaecol. 2020;66:28–40. 10.1016/j.bpobgyn.2019.12.002. 32014434

[10] Fraser IS. Non-contraceptive health benefits of intrauterine hormonal systems. Contraception. 2010;82(5):396–403. 10.1016/j.contraception.2010.05.005. 20933112

[11] Villavicencio J Allen RH. Unscheduled bleeding and contraceptive choice: increasing satisfaction and continuation rates. Open Access J Contracept. 2016;7:43–52. 10.2147/OAJC.S85565. 29386936 PMC5683158

[12] Jacobstein R. Liftoff: the blossoming of contraceptive implant use in Africa. Glob Health Sci Pract. 2018;6(1):17–39. 10.9745/GHSP-D-17-00396. 29559495 PMC5878070

[13] FHI 360, Society for Family Health Zambia (SFH), Population Services International (PSI), WCG Cares. *Final Report: Market Assessment for Potential Introduction of a New Hormonal IUCD in Zambia*. FHI 360/SFH/PSI/WCG Cares; 2016. Accessed August 15, 2021. https://www.hormonaliud.org/ourlibrary/Provider-Perspectives/resource/Zambia-Hormonal-IUS-Market-Assessment

[14] Danna K Jackson A Mann C Harris D. *Lessons Learned from the Introduction of the Levonorgestrel Intrauterine System (LNG-IUS) in Zambia and Madagascar*. WCG Cares/Population Services International; 2019. Accessed August 25, 2022. https://www.hormonaliud.org/ourlibrary/User-Perspectives/Current/EECO%3A-Lessons-Learned-from-the-Introduction-of-the-LNG-IUS-in-Zambia-and-Madagascar

[15] Routes2Results, Population Services International. Understanding the market potential of the IUS in Kenya and Nigeria. Final webinar. February 27, 2019. Accessed September 1, 2021. https://www.hormonaliud.org/ourlibrary/User-Perspectives/Potential/LEAP%3A-Hormonal-IUS-Market-Research-Qualitative-Findings-in-Kenya-and-Nigeria

[16] Routes2Results, Population Services International. Understanding end-user (women) and healthcare provider (HCP) preference for the IUS contraceptive in Nigeria and Kenya. Dissemination report. November 2019. Accessed September 1, 2021. https://www.hormonaliud.org/ourlibrary/User-Perspectives/Potential/LEAP%3A-Hormonal-IUS-Market-Research-Quantitative-Findings-in-Kenya-and-Nigeria

[17] Hormonal IUD Access Group. Hormonal IUD Access Group updates for key stakeholders. January 2022. Accessed August 7, 2021. https://www.hormonaliud.org/ourlibrary/Coordination-%26-Advocacy/Global/Hormonal-IUD-Access-Group-Updates-for-Key-Stakeholders

[18] Eva G Nanda G Rademacher K. Experiences with the levonorgestrel intrauterine system among clients, providers, and key opinion leaders: a mixed-methods study in Nigeria. Glob Health Sci Pract. 2018;6(4):680–692. 10.9745/GHSP-D-18-00242. 30591576 PMC6370358

[19] Brunie A Rademacher KH Nwala AA Danna K Saleh M Afolabi K. Provision of the levonorgestrel intrauterine system in Nigeria: provider perspectives and service delivery costs. Gates Open Research. 2020;4:119. 10.12688/gatesopenres.13135.1. 32908965 PMC7463110

[20] Sitrin D Pfitzer A Ndirangu G. Expanding contraceptive method choice with a hormonal intrauterine system: results from mixed methods studies in Kenya and Zambia. Glob Health Sci Pract. 2021;9(1):89–106. 10.9745/GHSP-D-20-00556. 33724921 PMC8087423

[21] Brunie A Stankevitz K Nwala AA. Expanding long-acting contraceptive options: a prospective cohort study of the hormonal intrauterine device, copper intrauterine device, and implants in Nigeria and Zambia. Lancet Glob Health. 2021;9(10):e1431–e1441. 10.1016/S2214-109X(21)00318-1. 34474001 PMC8440225

[22] Brunie A Nwala AA Stankevitz K. Factors affecting uptake of the levonorgestrel-releasing intrauterine device: a mixed-method study of social franchise clients in Nigeria. PLoS One. 2021;16(9):e0257769. 10.1371/journal.pone.0257769. 34587200 PMC8480829

[23] Brunie A Lydon M Stankevitz K. What are the prospects for the hormonal IUD in the public sector? A mixed-method study of the user population in Zambia. BMC Womens Health. 2022;22(1):178. 10.1186/s12905-022-01745-7. 35570281 PMC9107745

[24] Danna K Jaworski G Rahaivondrafahitra B. Introducing the hormonal intrauterine device in Madagascar, Nigeria, and Zambia: results from a pilot study. Reprod Health. 2022;6;19(1):4. 10.1186/s12978-021-01300-x. 34991651 PMC8734281

[25] Rademacher K. Applying the power of partnership to evaluation of a long-acting contraceptive. R&E Search for Evidence blog. May 8, 2017. Accessed March 15, 2022. https://researchforevidence.fhi360.org/applying-the-power-of-partnership-to-evaluation-of-a-long-acting-contraceptive

[26] Community of Practice on Method Choice, Hormonal IUD Access Group. Hormonal IUS updates: new insights and steps toward scale. Presented at: Global Technical Consultation – Day 1; June 25, 2020. https://www.hormonaliud.org/ourlibrary/User-Perspectives/resource/Hormonal-IUD-Updates%3A-June-2020-Technical-Consultation-%E2%80%93-Day-1

[27] Rowe P Farley T Peregoudov A IUD Research Group of the UNDP/UNFPA/WHO/World Bank Special Programme of Research; Development and Research Training in Human Reproduction. Safety and efficacy in parous women of a 52-mg levonorgestrel-medicated intrauterine device: a 7-year randomized comparative study with the TCu380A. Contraception. 2016;93(6):498–506. 10.1016/j.contraception.2016.02.024. 26916172 PMC5357727

[28] Laporte M Metelus S Ali M Bahamondes L. Major differences in the characteristics of users of the copper intrauterine device or levonorgestrel intrauterine system at a clinic in Campinas, Brazil. Int J Gynaecol Obstet. 2022;156(2):240–246. 10.1002/ijgo.13716. 33872406

[29] Todd CS Jones HE Langwenya N. Safety and continued use of the levonorgestrel intrauterine system as compared with the copper intrauterine device among women living with HIV in South Africa: a randomized controlled trial. PLoS Med. 2020;17(5):e1003110. 10.1371/journal.pmed.1003110. 32442189 PMC7244096

[30] Mirena. Prescribing information. Bayer HealthCare Pharmaceuticals Inc.; 2009. Accessed November 15, 2021. https://www.accessdata.fda.gov/drugsatfda_docs/label/2009/021225s027lbl.pdf

[31] Andersson K Odlind V Rybo G. Levonorgestrel-releasing and copper-releasing (Nova T) IUDs during five years of use: a randomized comparative trial. Contraception. 1994;49(1):56–72. 10.1016/0010-7824(94)90109-0. 8137626

[32] Chi IC. An evaluation of the levonorgestrel-releasing IUD: its advantages and disadvantages when compared to the copper-releasing IUDs. Contraception. 1991;44(6):573–588. 10.1016/0010-7824(91)90078-T. 1773615

[33] Nelson AL Massoudi N. New developments in intrauterine device use: focus on the US. Open Access J Contracept. 2016;7:127–141. 10.2147/OAJC.S85755. 29386944 PMC5683151

[34] About Mirena. Bayer. Accessed September 30, 2021. https://www.mirenahcp.com/

[35] About us. International Contraceptive Access Foundation. Accessed August 26, 2022. https://ica-foundation.org/ica-foundation/about-us/

[36] About the projects. International Contraceptive Access Foundation. Accessed August 26, 2022. https://ica-foundation.org/projects/about-the-projects/

[37] Population Council. *ICA Foundation Supports LNG IUS Access in Low- and Middle-Income Countries*. Population Council; 2020. Accessed September 1, 2021. 10.31899/ri1.1001

[38] Haelle T. Could the most effective birth control soon become the cheapest? Forbes Magazine. May 2, 2016. Accessed September 15, 2021. https://www.forbes.com/sites/tarahaelle/2016/05/02/could-the-most-effective-birth-control-soon-become-the-cheapest/?sh=1141a6f96853

[39] The Avibela Project. Medicines360. Accessed September 15, 2021. https://www.medicines360.org/avibela-project/

[40] FDA approves Medicines360’s LILETTA® (levonorgestrel-releasing intrauterine system) 52 mg to prevent pregnancy for up to six years. Press release. Medicines360. October 28, 2019. Accessed September 15, 2021. https://www.medicines360.org/2019/10/28/fda-approves-medicines360s-liletta/

[41] Nanda G Brennan C Brunie A. Adolescent girls’ and young women’s perspectives on the LNG-IUS in Nigeria. Final report presentation. May 2020. Accessed September 15, 2021. https://www.hormonaliud.org/ourlibrary/User-Perspectives/Youth/LEAP%3A-Adolescent-Girls’-and-Young-Womens’-Perspectives-on-the-LNG-IUS-in-Nigeria

[42] Nanda G Brennan C Brunie A. Adolescent girls’ and young women’s perspectives on the LNG-IUS in Zambia. Final report presentation. May 2020. Accessed September 15, 2021. https://www.hormonaliud.org/ourlibrary/User-Perspectives/Youth/LEAP%3A-Adolescent-Girls’-and-Young-Womens’-Perspectives-on-the-LNG-IUS-in-Zambia

[43] Homan R Rademacher KH Stankevitz K Brunie A. *Cost-effectiveness of Including the Hormonal IUD in the Contraceptive Method Mix in Nigeria and Zambia: Summary of Results*. Final brief. FHI 360/Population Services International; 2021. Accessed December 3, 2021. https://www.hormonaliud.org/ourlibrary/Cost-Effectiveness/resource/Cost-effectiveness-analysis-of-including-hormonal-IUD-in-the-contraceptive-method-mix-in-Nigeria-and-Zambia%3A-Summary-of-results

[44] Community of Practice on Method Choice, Hormonal IUD Access Group. Hormonal IUS updates: new insights and steps toward scale. Presented at: Global Technical Consultation – Day 2; June 26, 2020. https://www.hormonaliud.org/ourlibrary/User-Perspectives/resource/Hormonal-IUD-Updates%3A-June-2020-Technical-Consultation-%E2%80%93-Day-2

[45] Guttmacher Institute. *Adding It Up: Investing in Contraception and Maternal and Newborn Health in Nigeria*. Guttmacher Institute; 2019. Accessed August 26, 2022. https://www.guttmacher.org/fact-sheet/adding-it-up-contraception-mnh-nigeria

[46] Nigeria National Population Commission (NPC). *Nigeria Demographic and Health Survey 2018*. NPC/ICF; 2019. Accessed August 26, 2022. https://dhsprogram.com/pubs/pdf/FR359/FR359.pdf

[47] Commitment maker since 2012: Nigeria. FP2020. Accessed August 15, 2021. https://www.familyplanning2020.org/nigeria

[48] Nigeria. Federal Ministry of Health (FMOH). *Second National Strategic Health Development Plan: 2018–2022*. FMOH; 2018. Accessed September 15, 2021. https://www.health.gov.ng/doc/NSHDP%20II%20Final.pdf

[49] Nigeria. Federal Ministry of Health (FMOH). *Nigeria National Family Planning Blueprint 2020–2024*. FMOH; 2020. Accessed September 15, 2021. https://www.familyplanning2020.org/sites/default/files/Final-2020-Blueprint.pdf

[50] Projects: Nigeria. International Contraceptive Access Foundation. Accessed July 15, 2021. https://ica-foundation.org/projects/about-the-projects/nigeria/

[51] Commitment maker since 2015: Madagascar. FP2020. Accessed August 15, 2021. https://fp2030.org/madagascar

[52] Cain D. New Catalytic Opportunity Fund to support hormonal IUD introduction activities. January 19, 2021. Updated April 12, 2021. Accessed August 15, 2021. https://www.hormonaliud.org/post/new-catalytic-opportunity-fund-to-support-hormonal-ius-introduction-activities

[53] Introduction of long-acting family planning method to USAID and UNFPA product catalogs. FP2030. June 28, 2021. Accessed September 1, 2021. https://familyplanning2020.org/news/introduction-long-acting-family-planning-method-usaid-and-unfpa-product-catalogs

[54] Miranda L Townsend J Faúndes A Bahamondes L. The benefits and limitations of donating new contraceptive technology: the case of the International Contraceptive Access (ICA) Foundation and the LNG IUS Program in Brazil. Contraception. 2020;101(6):367–369. 10.1016/j.contraception.2020.01.003. 32007419

[55] Braun R Grever A. Scaling up access to implants: a summative evaluation of the Implants Access Program. Glob Health Sci Pract. 2020;8(2):205–219. 10.9745/GHSP-D-19-00383. 32467126 PMC7326518

[56] The Access Collaborative: putting self-injectable contraception within reach. PATH. November 2, 2020. Accessed September 1, 2021. https://www.path.org/articles/dmpa-sc-collaborative/

[57] PATH, Population Services International, FHI 360, Clinton Health Access Initiative, Population Council, WCG Cares. The highs, lows, and squishy middle of product introduction. Presented at: Global Health Science and Practice Technical Exchange; April 21–24, 2021. https://globalhealthxchange.com

[58] Shaping Equitable Market Access (SEMA) for Reproductive Health. Accessed September 1, 2021. https://www.semareprohealth.org/

[59] USAID Center for Accelerating Innovation and Impact (CII). *Healthy Markets for Global Health: A Market Shaping Primer*. CII; 2014. Accessed August 15, 2021. https://www.usaid.gov/sites/default/files/documents/1864/healthymarkets_primer.pdf

[60] Stout A Wood S Barigye G Kaboré A Siddo D Ndione I. Expanding access to injectable contraception: results from pilot introduction of subcutaneous depot medroxyprogesterone acetate (DMPA-SC) in 4 African countries. Glob Health Sci Pract. 2018;6(1):55–72. 10.9745/GHSP-D-17-00250. 29602866 PMC5878078

[61] Hubacher D Masaba R Manduku CK Veena V. Uptake of the levonorgestrel intrauterine system among recent postpartum women in Kenya: factors associated with decision-making. Contraception. 2013;88(1):97–102. 10.1016/j.contraception.2013.03.001. 23566383

[62] Hubacher D Masaba R Manduku CK Chen M Veena V. The levonorgestrel intrauterine system: cohort study to assess satisfaction in a postpartum population in Kenya. Contraception. 2015;91(4):295–300. 10.1016/j.contraception.2015.01.009. 25601351

[63] Nanda G Rademacher KH Solomon M Mercer S Wawire J Ngahu R. Experiences with the Levonorgestrel Intrauterine System (LNG-IUS) in Kenya: qualitative interviews with Mirena users and their partners. Eur J Contracept Reprod Health Care. 2018;10:1–6. 10.1080/13625187.2018.1499892. 30198796 PMC6191886

[64] Sedgh G Ashford LS Hussain R. *Unmet Need for Contraception in Developing Countries: Examining Women’s Reasons for Not Using a Method*. Guttmacher Institute; 2016. Accessed August 30, 2021. http://www.guttmacher.org/report/unmet-need-for-contraception-in-developing-countries

[65] Polis CB Hussain R Berry A. There might be blood: a scoping review on women’s responses to contraceptive-induced menstrual bleeding changes. Reprod Health. 2018;15(1):114. 10.1186/s12978-018-0561-0. 29940996 PMC6020216

[66] Rademacher KH Sergison J Glish L. Menstrual bleeding changes are NORMAL: proposed counseling tool to address common reasons for non-use and discontinuation of contraception. Glob Health Sci Pract. 2018;6(3):603–610. 10.9745/GHSP-D-18-00093. 30287535 PMC6172120

[67] Hoppes E Nwachukwu C Hennegan J. Global research and learning agenda for building evidence on contraceptive-induced menstrual changes for research, product development, policies, and programs. Gates Open Research. 2022;6:49. 10.12688/gatesopenres.13609.1. 35614964 PMC9114387

[68] Hubacher D. Clinical trial with the levonorgestrel intrauterine system to measure changes in hemoglobin and serum ferritin among anemic women in Kenya. NIH RePORTER. Project number: 1R01HD100497-01A1. Posted July 21, 2020. Accessed August 26, 2022. https://reporter.nih.gov/project-details/10050175

