# Supplementary material for: What Have We Learned? Implementation of a Shared Learning Agenda and Access Strategy for the Hormonal Intrauterine Device
Source: Glob Health Sci Pract. 2022 Oct 31;10(5):e2100789. doi: 10.9745/GHSP-D-21-00789 (PMC9622288; doi:10.9745/GHSP-D-21-00789)
Supplement: GHSP-D-21-00789-supplement.pdf [file GHSP-D-21-00789-supplement.pdf]

Supplement to: Rademacher KH, Sripipatana T, Danna K, et al. What have we learned? Implementation of a shared learning agenda and access strategy for the hormonal IUD. *Glob Health Sci Pract*. 2022;10(5):e2100789. https://doi.org/10.9745/GHSP-D-21-00789

Supplement. Revised Global Learning Agenda; Updated Through Consultation With Country-Level and Global Stakeholders in 2021

**Hormonal IUD Global Learning Agenda (2021 Version)**	**Current Status of Data From LMICs[a]**
1. Market potential	
a. What are the profile(s) of clients who will potentially use this method?	Some data exist - quantity/quality limited or insufficient
b. Is there or would there be demand for this method among sub-populations with high unmet need for FP (e.g., women in lower wealth quintiles, postpartum women, adolescents, WLHIV, PAC clients)?	Some data exist - quantity/quality limited or insufficient
c. What will demand for and uptake of the method likely be among different market segments?	Some data exist - quantity/quality limited or insufficient
d. What are reasons for demand/uptake among hormonal IUD users and how does this vary by different market segments?	Some data exist - quantity/quality limited or insufficient
e. Will introduction of the hormonal IUD help reach new FP users (i.e., current non-users)?	Some data exist - quantity/quality limited or insufficient
f. To what degree will introduction of the hormonal IUD result in “switching” and from what methods?	Some data exist - quantity/quality limited or insufficient
g. How does knowledge of non-contraceptive attributes of the hormonal IUD affect uptake? How does this vary by market segment?	Some data exist - quantity/quality limited or insufficient
h. What are the service delivery channel(s) where different sub-populations are mostly likely to seek LARC provision including the hormonal IUD?	Some data exist - quantity/quality limited or insufficient
i. Will introduction of the hormonal IUD lead to overall increases in contraceptive prevalence?	Data not currently available
2. Users' experiences with the method	
a. How do continuation rates of the hormonal IUD compare to continuation rates of other FP methods including LARCs?	Data exist
b. How do satisfaction rates with the hormonal IUD compare to continuation rates of other FP methods including LARCs?	Data exist
c. What specific attributes of the hormonal IUD do users find acceptable/unacceptable?	Some data exist - quantity/quality limited or insufficient

Supplement to: Rademacher KH, Sripipatana T, Danna K, et al. What have we learned? Implementation of a shared learning agenda and access strategy for the hormonal IUD. *Glob Health Sci Pract*. 2022;10(5):e2100789.
https://doi.org/10.9745/GHSP-D-21-00789

**Hormonal IUD Global Learning Agenda (2021 Version)**	**Current Status of Data From LMICs[a]**
d. What are users' perceptions of and experiences with menstrual changes associated with method use?	Some data exist - quantity/quality limited or insufficient
e. What are users' perceptions of and experiences with non-contraceptive clinical uses of method (e.g. treatment for heavy menstrual bleeding)?	Some data exist - quantity/quality limited or insufficient
f. What are perceptions of the method among partners and other key influencers/decision-makers in FP users' lives?	Some data exist - quantity/quality limited or insufficient
g. What are rates of hormonal IUD use disclosure to partners and other key influencers vs. rates of discreet use?	Data not currently available
h. What are typical expulsion rates for the hormonal IUD in LMIC settings? How does this compare to the copper IUD?	Some data exist - quantity/quality limited or insufficient
i. What are expulsion rates among women in the immediate or extended postpartum period in LMIC settings? (Also, see section 6 below)	Some data exist - quantity/quality limited or insufficient
3. Providers' experiences with the method	
a. What are health care providers' perceptions of and experiences with the hormonal IUD?	Data exist
b. What are health care providers' understanding of potential complications with the method and their ability to manage these?	Data not currently available
c. What provider-side barriers are there to method provision? How can these be effectively addressed?	Some data exist - quantity/quality limited or insufficient
d. What are providers' perceptions of menstrual changes associated with method use and how do/will they counsel on these?	Some data exist - quantity/quality limited or insufficient
e. What are providers' perceptions of other side effects associated with method use and how do/will they counsel on these?	Some data exist - quantity/quality limited or insufficient
f. What are providers' perceptions of non-contraceptive clinical uses of the method (e.g., treatment for heave menstrual bleeding)?	Some data exist - quantity/quality limited or insufficient
g. How do providers' perceptions and experiences vary by level of pre-existing experience with other LARCs?	Data not currently available

Supplement to: Rademacher KH, Sripipatana T, Danna K, et al. What have we learned? Implementation of a shared learning agenda and access strategy for the hormonal IUD. *Glob Health Sci Pract*. 2022;10(5):e2100789. https://doi.org/10.9745/GHSP-D-21-00789

**Hormonal IUD Global Learning Agenda (2021 Version)**	**Current Status of Data From LMICs[a]**
4. Service delivery – training and method provision	
a. What are cost-effective approaches for training on the hormonal IUD for LARC-experienced providers?	Some data exist - quantity/quality limited or insufficient
b. What are cost-effective approaches for training on the hormonal IUD for LARC-inexperienced providers?	Some data exist - quantity/quality limited or insufficient
c. What level of post-training supervision is required? How does this differ among LARC-experienced vs. inexperienced providers?	Data not currently available
d. What level of training/refresher training is required to transition to different inserter types for various hormonal IUD products?	Data not currently available
e. What are effective service delivery models/channels for hormonal IUD provision? How does this differ by context?	Some data exist - quantity/quality limited or insufficient
f. To what extent is comprehensive counseling offered (including on side effects, menstrual changes, sexually transmitted infection prevention, options for removal, etc.)?	Some data exist - quantity/quality limited or insufficient
g. What are new or existing opportunities for task-sharing to support hormonal IUD service provision	*Apply existing knowledge from provision of other LARCs
h. What challenges, if any, do providers commonly experience when providing the hormonal IUD and how can these be overcome?	Data not currently available
5. Service delivery – demand creation	
a. What are effective demand creation strategies for the hormonal IUD to reach different market segments?	Some data exist - quantity/quality limited or insufficient
b. How should messaging about the method's attributes (contraceptive and noncontraceptive) be incorporated into demand creation strategies?	Some data exist - quantity/quality limited or insufficient
c. How can messaging about the method be integrated into other health services? (Also, see Section 6 below)	Data not currently available
d. How can messaging about the method be integrated into other sectors (e.g., via menstrual health platforms)?	Data not currently available

Supplement to: Rademacher KH, Sripipatana T, Danna K, et al. What have we learned? Implementation of a shared learning agenda and access strategy for the hormonal IUD. *Glob Health Sci Pract*. 2022;10(5):e2100789. https://doi.org/10.9745/GHSP-D-21-00789

**Hormonal IUD Global Learning Agenda (2021 Version)**	**Current Status of Data From LMICs[a]**
6. Service delivery – integrated approaches*	
a. How can we reach WLHIV with LARCs including hormonal IUD (e.g. through integrated FP-HIV service delivery models)?	Some data exist - quantity/quality limited or insufficient
b. How can we reach women in the immediate and extended postpartum period with LARCs including hormonal IUD?	Some data exist - quantity/quality limited or insufficient
c. How can we integrate LARC services including hormonal IUD into youth-friendly FP programs?	*Apply existing knowledge from provision of other LARCs
d. How can we integrate LARC services including hormonal IUD provision into PAC services?	*Apply existing knowledge from provision of other LARCs
e. How can we integrate LARC services including hormonal IUD with cervical cancer screening?	*Apply existing knowledge from provision of other LARCs
f. How can we integrate LARC counseling/services including hormonal IUD with child immunization programs?	*Apply existing knowledge from provision of other LARCs
g. How can we offer LARC services including hormonal IUD in humanitarian and refugee settings?	*Apply existing knowledge from provision of other LARCs
h. How can we reach women in the antenatal period with counseling/information on LARCs including hormonal IUD?	*Apply existing knowledge from provision of other LARCs
7. Service delivery – removal	
a. How can countries ensure reliable access to affordable hormonal IUD removal services?	Data not currently available
b. In addition to desire for pregnancy, what are reasons for seeking removal services?	Some data exist - quantity/quality limited or insufficient
c. What is the prevalence of difficult removals and what are common challenges providers experience?	Data not currently available
d. Is self-removal an effective, safe, and acceptable option for women to try in LMIC settings? If yes, which group(s) of women?	Data not currently available
8. Noncontraceptive attributes	
a. Can use of the hormonal IUD help prevent or treat iron-deficiency anemia?	Some data exist - quantity/quality limited or insufficient

Supplement to: Rademacher KH, Sripipatana T, Danna K, et al. What have we learned? Implementation of a shared learning agenda and access strategy for the hormonal IUD. *Glob Health Sci Pract*. 2022;10(5):e2100789. https://doi.org/10.9745/GHSP-D-21-00789

**Hormonal IUD Global Learning Agenda (2021 Version)**	**Current Status of Data From LMICs[a]**
b. Does scale-up of the hormonal IUD help reduce rates of anemia?	Data not currently available
c. Can scale-up of the hormonal IUD help promote women's health in other areas? [specify indicators]	Some data exist - quantity/quality limited or insufficient
9. Cost-effectiveness and pricing	
a. To what extent is the hormonal IUD cost-effective for clients compared to other FP methods including other LARCs?	Data exist
b. To what extent is the hormonal IUD cost-effective for donors/procurers compared to other FP methods including other LARCs?	Data exist
c. What is the willingness-to-pay for the hormonal IUD among different market segments and in different channels?	Some data exist - quantity/quality limited or insufficient
d. Does use of the hormonal IUD reduce financial or opportunity costs (e.g., through savings on menstrual hygiene supplies, days of school/work, etc.)	Some data exist - quantity/quality limited or insufficient
e. What is willingness-to-stock/pay/procure among different purchasers and sectors (e.g., public vs. private)?	Data not currently available
f. What are the estimated costs / cost-savings for scaling-up the hormonal IUD as part of the national or sub-national contraceptive method mix?	Some data exist - quantity/quality limited or insufficient
10. Equity	
a. What populations are being reached with initial/early roll-out of the hormonal IUD?	Some data exist - quantity/quality limited or insufficient
b. How can we ensure equitable access to LARC services across market segments for traditionally underserved populations?	*Apply existing knowledge from provision of other LARCs
11. Country dynamics for scale-up	
a. To what extent is the Ministry of Health ready to scale the hormonal IUD in the public sector? What criteria impact decision-making?	*Also refer to country-level monitoring indicators[b]
b. To what extent are private sector actors contributing to scaling the hormonal IUD? What factors encourage or discourage their participation?	*Also refer to country-level monitoring indicators[b]

Supplement to: Rademacher KH, Sripipatana T, Danna K, et al. What have we learned? Implementation of a shared learning agenda and access strategy for the hormonal IUD. *Glob Health Sci Pract*. 2022;10(5):e2100789. https://doi.org/10.9745/GHSP-D-21-00789

**Hormonal IUD Global Learning Agenda (2021 Version)**	**Current Status of Data From LMICs[a]**
c. What is the role of civil society in supporting and sustaining scale-up of the hormonal IUD as part of a broader method mix?	*Also refer to country-level monitoring indicators[b]
d. What are unique considerations / challenges for supply chain management for this method? How can these be addressed?	*Also refer to country-level monitoring indicators[b]

Abbreviations: FP, family planning; IUD, intrauterine device; LARC, long-acting reversible contraceptive; LMICs, low- and middle-income countries; PAC, postabortion care; WLHIV, women living with HIV.
[a] Assessment of the current status of evidence based on co-authors' review and assessment of existing data from across countries. See Table 3 for additional details.
[b] Country-level indicators monitored by members of the Hormonal IUD Access Group; for more information, email info@hormonaliud.org.
